# Supplementary material for: Plant survival and keystone pollinator species in stochastic coextinction models: role of intrinsic dependence on animal-pollination
Source: Sci Rep. 2017 Jul 31;7:6915. doi: 10.1038/s41598-017-07037-7 (PMC5537349; doi:10.1038/s41598-017-07037-7)

Supplementary Information

Plant survival and keystone pollinator species in stochastic coextinction models: role of intrinsic dependence on animal-pollination

Anna Traveset[1], Cristina Tur[1] and Víctor M. Eguíluz[2*]

[1]Institut Mediterrani d'Estudis Avançats IMEDEA (CSIC-UIB), Terrestrial Ecology Group. C/ Miquel Marqués 21, E07190-Esporles, Mallorca, Balearic Islands, Spain

[2] Instituto de Física Interdisciplinar y Sistemas Complejos IFISC (CSIC-UIB), E07122-Palma de Mallorca, Spain

*Corresponding author: victor@ifisc.uib-csic.es

Calculation of the survival probability of plants

At each extinction event one pollinator is selected at random for extinction. The probability that plant *I* survives is given by

$$P_{ij} = 1 - IPD_i d_{ij} \ , \tag{S1}$$

where IPD_i is the dependence on pollinators of plant *i*. Averaging over realizations leads to the expected surviving probability of plant *i*,

$$P_i(1) = \langle P_{ij} \rangle = 1 - IPD_i \langle d_{ij} \rangle = 1 - \frac{IPD_i}{N_P} \ , \tag{S2}$$

where N_p is the number of pollinators. For the second extinction, we select a random pollinator *k* from the surviving set. Assuming that the first extinction has not led to tertiary extinctions, the probability of plant *i* to survive to 2 extinctions is given by

$$P_i(2) = \langle \left(1 - IPD_i d_{ij}\right)(1 - IPD_i d'_{ik}) \rangle = \left(1 - \frac{IPD_i}{N_P}\right)\left(1 - \frac{IPD_i}{N_P - 1}\right) . \tag{S3}$$

For *e* extinctions we can obtain

$$P_i(e) = \langle \left(1 - IPD_i d_{ij}\right)(1 - IPD_i d'_{ik}) \rangle = \left(1 - \frac{IPD_i}{N_P}\right)\left(1 - \frac{IPD_i}{N_P - 1}\right) \dots \left(1 - \frac{IPD_i}{N_P - e + 1}\right). \tag{S4}$$

For plants with $IPD_i \neq 1$, this can be expressed as

$$P_i(e) = \frac{\Gamma(N_P + 1 - IPD_i)}{\Gamma(N_P + 1 - IPD_i - e)} \frac{\Gamma(N_P + 1 - e)}{\Gamma(N_P + 1)} \ , \tag{S5}$$

where Γ(.), is the Gamma function; while for IPD_i=1,

$$P_i(e) = \frac{N_P - e}{N_P} \ . \tag{S6}$$

Supplementary Figure S1 shows the comparison between the numerical and the theoretical values. For the theoretical values the fraction of surviving plants is obtained

using Equation (S4) for the empirical values of SB (top) and PM (bottom). The discrepancies can be explained by the ansatz of absence of pollinator extinctions after a plant extinction, which depends on the connectance of the plant-pollinator network.

Supplementary Figure S1. Fraction of surviving plants as a function of the fraction of extinction events. The numerical response (triangles) is compared with the theoretical value (squares) for the complete model (model F) and for the case where all the IPD values are set to 1 (model D), (a) SB site and (b) PM site. The theoretical curves are obtained averaging Equations (S5) and (S6) with the empirical IPD values.

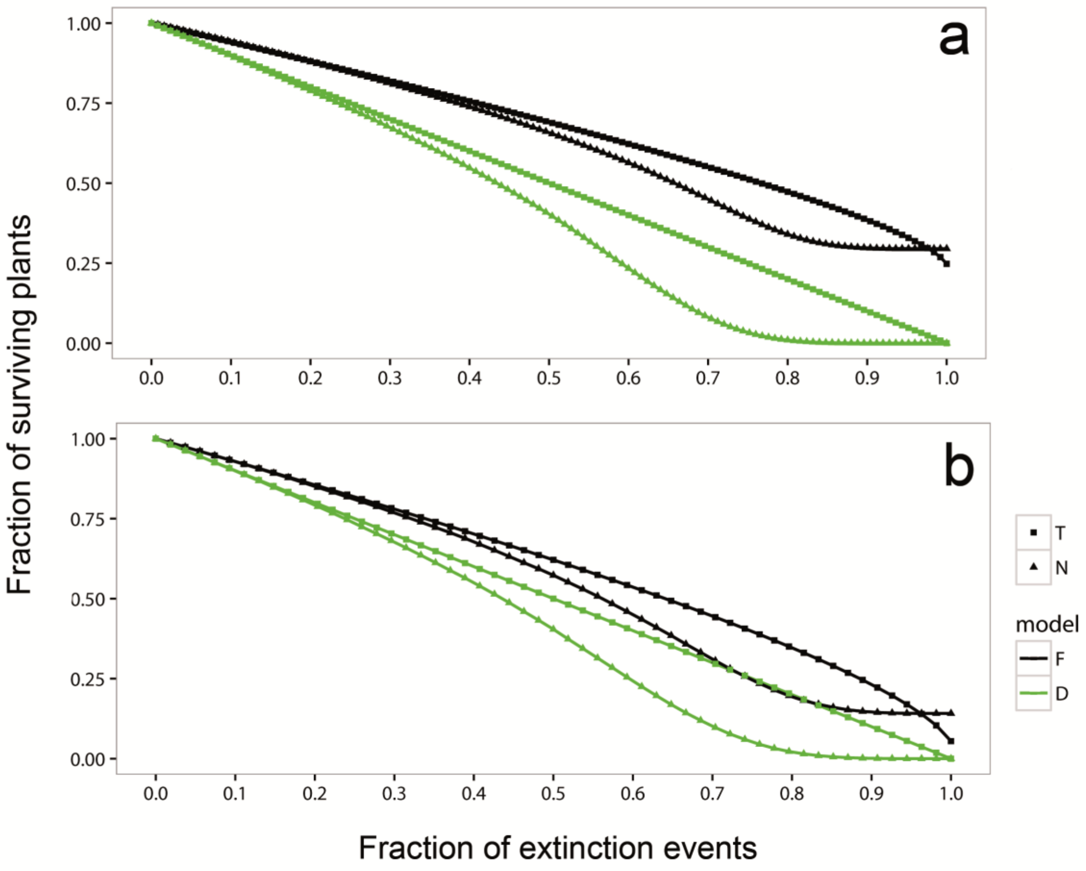

Supplementary Figure S2. Plant robustness *vs.* extinction models. Mean and 95% confidence intervals of plant robustness estimated both with the topological coextinction model (TCM) and the stochastic coextinction model (SCM) in the two communities (a: Son Bosc, b: Puig Major) under the three pollinator removal scenarios (R: random, G: generalist, S: specialist). Consistent significant differences were found between the two models in all scenarios.

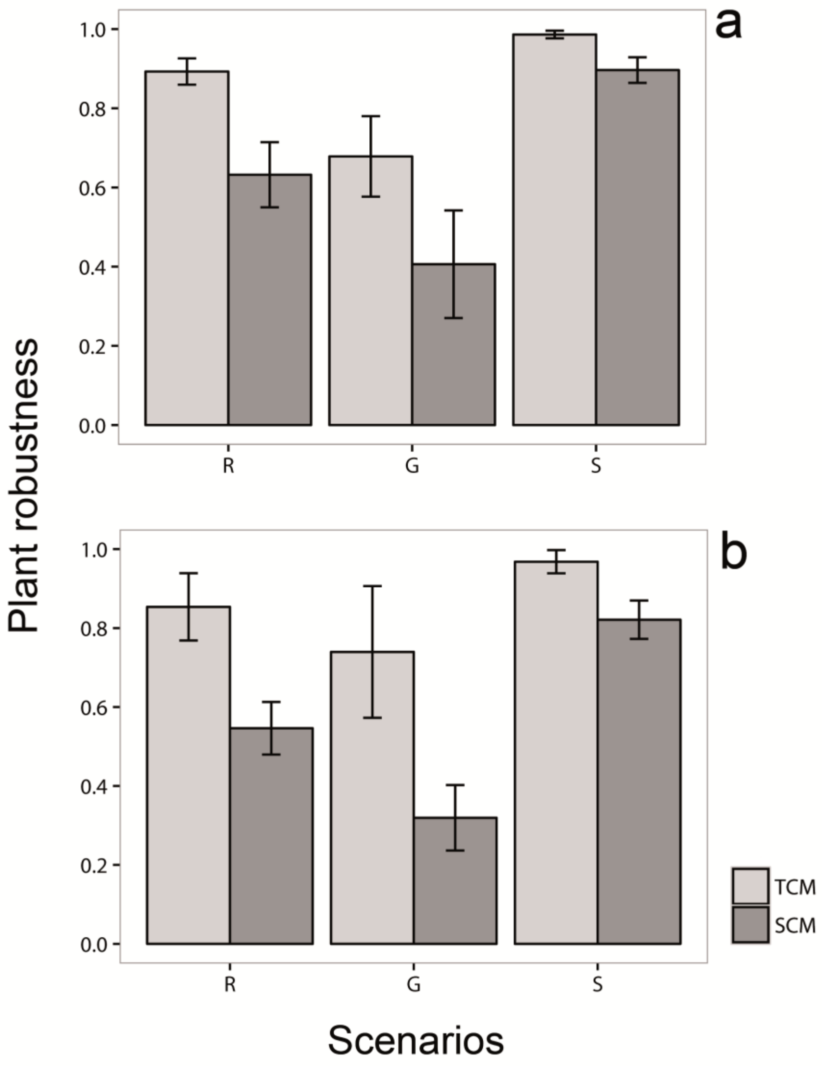

Supplementary Figure S3. Plant robustness *vs.* degree and pollinator dependence. Difference in plant robustness among the topological coextinction model (TCM) and the stochastic coextinction model (SCM) according to: (a) plant degree, and (b) plant dependence on insect pollinators (IPD). Each dot is a plant species and colours indicate the sampling community. For plants above the line, robustness was higher when estimated with TCM than with SCM. TCM tends to underestimate robustness for plants with few interactions (small degree) and plants with a low dependence on pollinators to produce seeds.

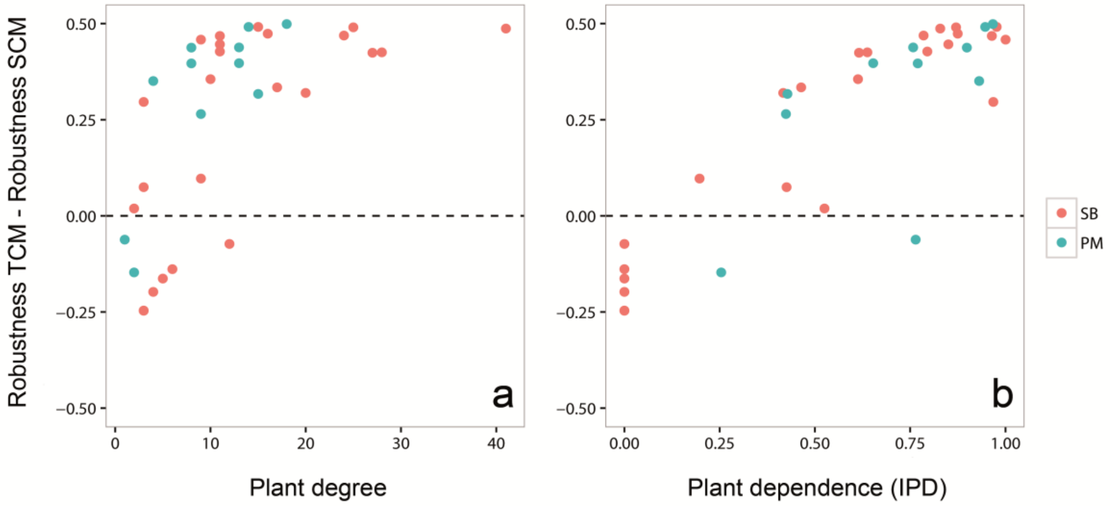

Supplementary Figure S4. Surviving species depending on extinction scenario. Fraction of surviving species (a,c) and surviving plant species (b,d) resulting from pollinator extinctions with the full model under the three extinction scenarios in SB (above) and PM (below) communities. R: random scenario, extinctions occur randomly across pollinators; G: generalist scenario, extinctions go sequentially from the most to the least linked species; S: specialist scenario, extinctions go sequentially from the least to the most linked species.

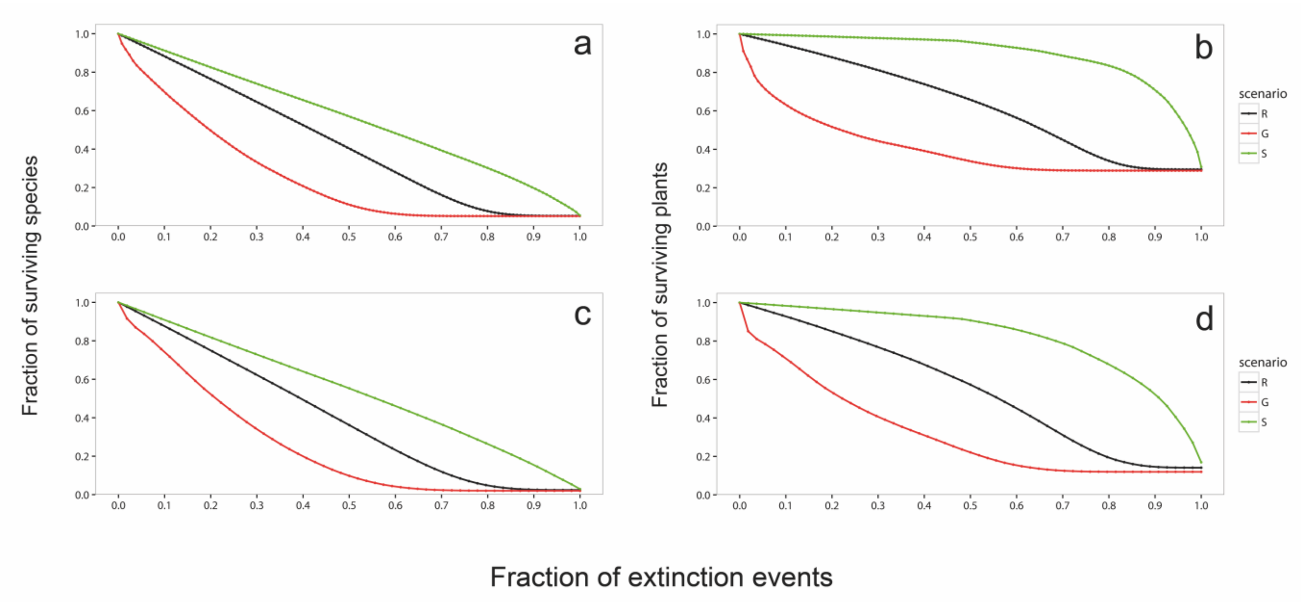

Supplement: Supplementary file 1 — Supplementary Information [file 41598_2017_7037_MOESM1_ESM.pdf]
